# Reducing Diagnostic Delays in Head and Neck Cancer Through Surgeon-Performed Ultrasound-Guided Fine-Needle Aspiration and Same-Day Cytology Results

**DOI:** 10.7759/cureus.109834

**Published:** 2026-05-28

**Authors:** Alex Berg Kristensen, Anne Fog Lomholt, Tina Klitmoeller Agander, Tobias Todsen

**Affiliations:** 1 Department of Otorhinolaryngology, Head and Neck Surgery and Audiology, Rigshospitalet, Copenhagen, DNK; 2 Department of Clinical Medicine, University of Copenhagen, Copenhagen, DNK; 3 Department of Pathology, Rigshospitalet, Copenhagen, DNK; 4 Department of Applied Mathematics and Computer Science, Technical University of Denmark, Kongens Lyngby, DNK

**Keywords:** cancer of the head and neck, cervical neck masses, surgeon-performed ultrasound, ultrasound-guided biopsy, ultrasound-guided fine-needle aspiration

## Abstract

Purpose: There is increasing interest among ear, nose, and throat (ENT) surgeons in performing ultrasound and ultrasound-guided fine-needle aspiration biopsy (US-FNAB). Surgeon-performed ultrasound has the potential to streamline the diagnostic pathway and reduce waiting times for patients evaluated for suspected head and neck cancer. However, concerns have been raised about diagnostic accuracy when surgeons, rather than radiologists, perform US-FNAB. This study aimed to assess the diagnostic quality and time to diagnosis of surgeon-performed US-FNAB with same-day cytology for all patients referred to an academic head and neck cancer outpatient clinic.

Methods: This retrospective study reviewed patients’ records from a fast-track head and neck cancer outpatient clinic. All patients underwent surgeon-performed US-FNAB with same-day final cytological evaluation by a dedicated cytopathologist. Data included demographics, biopsy site, cytology and histology results, and time from referral to a diagnostic plan. The primary outcome was the inadequacy rate of US-FNAB from cytology reports, while the secondary outcomes included the time to diagnostic plan and diagnostic accuracy. The time to the diagnostic plan was defined as the time from referral to the establishment of a final diagnostic plan. Diagnostic accuracy was assessed using US-FNAB results and final histology diagnoses when available.

Results: A total of 155 US-FNAB samples from 117 patients were analyzed. The overall inadequacy rate was 11%, with the highest rate observed in thyroid lesions (five of 23; 21.7%). No significant difference was found between residents (5.6%) and consultants (15.3%) (p=0.054). Same-day cytology results were available for 114 (97.4%) patients. A diagnostic plan was established at the initial visit in 71 (60.7%) cases, while the remaining patients had a median time to diagnostic plan of 11.5 days (IQR 8-16). The overall median time from referral to diagnostic plan was six days (IQR 4-16). Histopathological correlation was available for 62 (53%) patients. The risk of malignancy was 0% for benign, 45.5% for undetermined, 29.4% for suspicious for malignancy, and 85.2% for malignant cytology.

Conclusion: Surgeon-performed US-FNABs with same-day cytology achieved high adequacy rates, allowing most patients to receive a diagnostic plan during their initial visit. Residents performed similarly to consultants, supporting the feasibility of integrating US-FNAB into routine practice across different experience levels.

## Introduction

Ultrasound-guided fine-needle aspiration biopsy (US-FNAB) is a cornerstone of the diagnostic workup for most head and neck lesions [1]. Traditionally, patients seen in a head and neck clinic with neck lumps were referred to radiology departments for US-FNAB. However, previous studies indicate that waiting time for US-FNAB introduces diagnostic delays for patients suspected of head and neck cancer (HNC) [2,3]. As treatment delay may negatively affect the prognosis of HNC, it is crucial to ensure a fast diagnostic workup [4]. Diagnostic head and neck ultrasound is increasingly performed by ear, nose, and throat (ENT) surgeons in an office-based setting as an extension of the clinical examination and for ultrasound-guided interventions [5-8]. This also allows surgeons to perform US-FNAB during the patient’s initial visit, thereby reducing the number of clinical visits needed to establish a diagnosis [9-11]. However, previous studies have used highly experienced and selected US-trained head and neck consultants to perform US-FNAB in fast-track clinics [10], and it is unknown whether the diagnostic results can be generalized to settings in which surgeon-performed US-FNAB is standard clinical practice in the department, including US-FNAB by residents [12,13]. Another important reason for diagnostic delay is inadequate FNAB samples and waiting time for cytology diagnosis. To decrease the number of inadequate FNABs, rapid on-site evaluation (ROSE) has become popular, as FNAB smears are immediately assessed microscopically at the point of care to determine adequacy and decide whether repeat procedures are needed on the same visit. Although ROSE can significantly decrease the rate of inadequate results for both radiologists and surgeons, there is still a waiting time for the final cytology diagnosis [14]. At the HNC clinic at Rigshospitalet, Copenhagen, Denmark, we have instead implemented a same-day cytology service in which final cytological diagnoses are provided within two hours from sampling. In this setting, US-FNAB is performed daily in the outpatient clinic as an extension of the clinic exam by both ENT residents and consultants, and they receive the cytology diagnosis by phone from a dedicated cytopathologist. The final diagnosis can thereby be established during the first visit, and an additional biopsy can be performed the same day if FNAB is inadequate. However, whether this fast-track concept for an HNC clinic, in which surgeon-performed US-FNAB and same-day cytology are implemented in daily clinical practice, improves the diagnostic pathway has not yet been explored. This study aims to examine the rate of inadequate results and the time to diagnosis in a setting where US-FNAB is performed as an extension of the clinical examination by both ENT residents and consultants with a same-day cytology service available.

## Materials and methods

We conducted a retrospective study of patients’ records from a fast-track HNC outpatient clinic at Rigshospitalet, Copenhagen University Hospital, Denmark. The study received institutional ethics approval from Rigshospitalet (ID number 23030397), and retrospective data were collected from patient records by a single reviewer (ABK). This study includes all patients who underwent US-FNAB from the 15th of May to the 30th of June 2022 at the fast-track HNC outpatient clinic at Rigshospitalet.

The fast-track HNC outpatient clinic at Rigshospitalet received referrals of patients suspected of HNC from private practice ENT surgeons, other hospital departments, and general practitioners (GPs) through the Danish fast-track cancer system [15]. In the fast-track outpatient clinic, each patient underwent a comprehensive ENT examination, including fiberoptic endoscopy and surgeon-performed neck ultrasound. In Denmark, head and neck ultrasound is part of ENT residency training, and ENT residents are expected to perform US-FNAB after the third year of training, having completed formal ultrasound training and certification [5,16,17]. Both consultants and fourth- and fifth-year ENT residents were conducting diagnostic workups for patients referred to the outpatient cancer clinic at Rigshospitalet. Here, they would perform a diagnostic ultrasound as an extension of the clinical exam and perform US-FNAB if a suspicious lesion was identified. An ultrasound machine was available in every consultation room, and the surgeon could therefore perform US-FNAB during the same consultation if needed, without any waiting time. The surgeon would prepare a smear from the US-FNAB sample, which was air-dried and sent to the pathology department for May-Grünwald-Giemsa (MGG) staining without the use of ROSE. The slides were immediately evaluated by a dedicated head and neck cytopathologist at the pathology department, who reported the cytological findings to the surgeon by phone within two hours, followed by a formal written report. The aim was to provide a clinically relevant cytopathological classification of the US-FNAB samples within two hours, enabling the surgeon to immediately plan treatment or further diagnostic procedures for the patient. The patients were therefore booked in the morning and told to wait for the cytology result so US-FNAB could be repeated during the same visit if the sample was inadequate.

Cytopathological classification of US-FNAB

Variables extracted from the patient chart included patient demographics and clinical characteristics (anatomical region, side, and ultrasound findings), biopsy details, cytology and histology reports, and the time from referral to establishment of the final diagnostic plan. The primary outcome was the cytology inadequacy rate of US-FNAB biopsies. Criteria for inadequacy included insufficient cellularity, lack of representative material, excessive blood or colloid contamination, or poor or incorrect smear technique. The US-FNAB smears were categorized as either inadequate or adequate FNA smears suitable for cytological diagnosis. This study does not include a control group. Instead, the observed inadequacy rates are compared with previously published data reporting inadequacy rates for both surgeon- and radiologist-performed US-FNAB, which serve as reference benchmarks.

Secondary outcomes included time to diagnostic plan and the US-FNAB diagnostic accuracy. The time to a diagnostic plan was defined as the time from referral until either an operation booking was made, a multidisciplinary team (MDT) decision was made, or patients were discharged with a benign diagnosis not requiring further intervention. The diagnostic accuracy of US-FNAB results was calculated using the final histologic diagnosis, when available. Concordant benign cytology and histology were considered “true negatives.” Cytology samples categorized as malignant were considered “true positives” when subsequent histological examination confirmed malignancy, irrespective of whether a specific histologic subtype was identified in the cytology report. If a benign cytology result was not followed by histology within six months, the sample was classified as benign, consistent with routine clinical practice, as a benign US-FNAB rarely yields a histology specimen. Diagnostic adequacy of US-FNAB samples was assessed according to established diagnostic systems: salivary glands by the Milan system, thyroid glands by a Danish national classification (comparable to Bethesda with a separate cyst category), with the category “undetermined” corresponding to group III and IV. Lymph nodes are categorized by a modified version of the Sydney system, with limited ancillary techniques available [18-20]. All of the above-mentioned systems include an inadequate/non-diagnostic category; therefore, in this study, these were grouped into a common inadequate category. Diagnostic categories, regardless of topography, were overall grouped into (1) malignant, (2) suspicious for malignancy, (3) diagnostically undetermined, and (4) benign. Each category has been explained in the Appendices. The risk of malignancy (ROM) was calculated for each diagnostic category. Re-biopsies and additional biopsies from other lesions/sites were not included in the analysis.

Statistics

The primary outcome was the inadequacy rate of US-FNAB and will be reported as a number and percentage of all samples performed n (%). The secondary outcomes include time to diagnostic plan and diagnostic accuracy. Time to diagnostic plan will be reported as the median time in days and interquartile range (IQR) (Q1-Q3), whereas diagnostic accuracy will be reported as a percentage. The adequacy of US-FNABs was compared between consultants and residents using binary logistic regression, with adequate/inadequate as the outcome. Sensitivity, specificity, and diagnostic accuracy of US-FNAB were calculated by comparing initial cytology with corresponding histology. Inadequate, undetermined, and suspected malignant samples were excluded from the sensitivity and specificity analyses. A two-tailed p-value < 0.05 was considered statistically significant. All analyses were performed using R version 4.2.2 (R Foundation for Statistical Computing, Vienna, Austria).

## Results

This study reviewed medical records from 117 patients who underwent US-FNAB in the fast-track HNC outpatient clinic at Rigshospitalet between 15th May and 30th June 2022. Of 117 patients, 24 (20.5%) underwent more than one biopsy, yielding 155 surgeon-performed US-FNAB samples for analysis. The median age was 61 years (IQR 48-74), and 63 (53.8%) were male (Table 1). Patients referred to the HNC fast-track outpatient clinic mainly originated from private ENT surgeons (69, 59%), other hospital departments (34, 29.1%), but also included seven (6%) from other hospitals with an ENT department, and five (4.3%) from GPs. All US-FNAB procedures were performed by surgeons, and none of the patients were referred to the radiology department for US-FNAB. Cytology results (diagnosis) were available on the same day for 114 (97.4%) patients.

**Table 1:** Demographics of the 117 included patients. Years and days are reported as median (IQR (Q1-Q3)) and values reported as n (%). ENT: ear, nose, and throat, GP: general practitioner, HNC: head and neck cancer, RH: Rigshospitalet

Demographics	Values
Age (years)	61 (48-74)
Gender (male)	63 (53.8%)
Referred from	
Private ENT	69 (59%)
Other hospital department	36 (30.8%)
ENT from other hospitals	7 (6%)
GP	5 (4.3%)
Visits to the HNC clinic	
First visit	97 (82.9%)
Relapsing or control patients	13 (11.1%)
Previous visit(s) at another ENT department	7 (6%)
Days from referral to appointment at RH	4 (2-6)
Days from referral to diagnostic plan	6 (4-16)
Previous cancer	
HNC	15 (12.8%)
Other cancer	18 (15.4%)
Anti-thrombotic medication	17 (14.5%)

In total, nine residents and 13 consultants performed 155 US-FNAB procedures in 117 patients, and 24 (20.5%) patients underwent more than one biopsy during their initial visit. Of the 24 (20.5%) patients, 10 (8.5%) required a repeat biopsy. Among these, six (60%) re-biopsies were classified as inadequate and four (40%) as benign. Seventy-one (60.7%) patients received a diagnostic plan during their initial fast-track outpatient clinic visit, whereas the remaining 46 (39.3%) had a longer median time to diagnostic plan of 11.5 days (IQR 8-16). The median time to a diagnostic plan from referral was six days (IQR 4-16) for all patients.

Of the 155 surgeon-performed US-FNAB samples, 17 (11%) were inadequate, and 138 (89%) were adequate (Table 2). Among the adequate samples, 73 (47.1%) were classified as benign, 12 (7.7%) as undetermined, 18 (11.6%) as suspected malignant, and 35 (22.6%) as malignant. Inadequacy rates by site were 21.7% in the thyroid gland, 10% in salivary glands, and 6.8% in lymph nodes. Re-biopsies were performed in 10 cases. In three of those cases, both the primary and the re-biopsy were inadequate.

**Table 2:** Distribution of US-FNABs in the head and neck region of the 117 patients with a total of 155 US-FNABs performed from the 15th of May to the 30th of June 2022 by 22 physicians. Values reported n (%). *Other includes lumps in the neck, axillary lymph nodes, lip, oral cavity, and nose. US-FNAB: ultrasound-guided fine-needle aspiration biopsy

	Inadequate	Benign	Undetermined	Suspected malignant	Malignant	Total
Thyroid	5 (21.7%)	6 (26.1%)	4 (17.4%)	3 (13%)	5 (21.7%)	23 (14.8%)
Salivary glands	4 (10%)	23 (57.5%)	0	10 (25%)	3 (7.5%)	40 (25.8%)
Lymph nodes	5 (6.8%)	37 (50.7%)	3 (4.1%)	3 (4.1%)	25 (34.2%)	73 (47.1%)
Neck cysts	0	5 (62.5%)	3 (37.5%)	0	0	8 (5.2%)
Other*	3 (27.3%)	2 (18.2%)	2 (18.2%)	2 (18.2%)	2 (18.2%)	11 (7.1%)
Total of all samples	17 (11%)	73 (47.1%)	12 (7.7%)	18 (11.6%)	35 (22.6%)	155 (100%)

Histopathological correlation was available in 62 (53%) patients, comprising 29 (46.8%) benign and 33 (53.2%) malignant samples. The histopathological results from samples categorized as inadequate, undetermined, and suspected malignant were excluded from the diagnostic accuracy analysis (Table 3). Compared with final histopathology, cytology correctly identified 23 (37.1%) true-positive samples, 12 (19.4%) true-negative samples, no false-negative samples, and two (3.2%) false-positive samples. The overall diagnostic accuracy of adequate US-FNAB was 94.6%, with a sensitivity of 100% and a specificity of 85.7%. The two false-positive cases included a salivary gland tumor interpreted as a giant cell tumor and a thyroid nodule with benign thyroid tissue on histology.

**Table 3:** The risk of malignancy (ROM) of the 117 patients and a total of 62 histopathology samples. Values reported n (%). Histology collected: eight core needle biopsies, three forceps biopsies, and 51 surgical specimens. US-FNAB: ultrasound-guided fine-needle aspiration biopsy

	Patients with US-FNAB	Benign histopathology	Malignant histopathology	ROM
Inadequate	9 (7.7%)	3 (10.3%)	0 (0%)	0%
Benign	53 (45.3%)	12 (41.4%)	0 (0%)	0%
Undetermined	11 (9.4%)	2 (6.9%)	5 (15.2%)	45.5%
Suspected malignant	17 (14.5%)	10 (34.5%)	5 (15.2%)	29.4%
Malignant	27 (23.1%)	2 (6.9%)	23(69.7%)	85.2%
Total	117	29 (46.8%)	33 (53.2%)	-

The ROM was calculated for all diagnostic categories among the 117 patients (Table 3). The ROM was 0% for benign samples, 45.5% for undetermined samples, 29.4% for suspected malignant samples, and 85.2% for malignant samples.

A subgroup analysis of sample adequacy showed that nine residents performed 72 US-FNABs (46.5%), and 13 consultants performed 83 US-FNABs (53.5%). Inadequate samples were observed in 4/72 (5.6%) procedures by residents and 13/83 (15.7%) procedures by consultants (p=0.054).

## Discussion

Surgeon-performed US-FNAB, integrated with same-day cytology, proved feasible and effective for the outpatient evaluation of head and neck lesions. Adequate sample rates were high (89%), and most patients (71, 60.7%) received a diagnostic plan on the day of the initial visit, with a median time of six days (IQR 4-16) from referral to diagnostic plan. ENT residents with formal ultrasound training performed comparably to consultants, indicating that the procedure could be successfully implemented across operators with varying levels of experience.

This is one of the first studies to explore how a clinical setting in which surgeon-performed US-FNAB is integrated with same-day cytology is fully implemented, rather than radiologist-performed US-FNAB. Additionally, the samples were obtained from all head and neck subsites and represented diverse pathologies, rather than being limited to thyroid lesions, as in other studies [3,21-24].

A strength of this study in terms of implementing US-FNAB in a clinical setting is that the US-FNAB procedure was performed by ENT surgeons with different levels of experience, including both residents and consultants, supporting the generalizability of the findings to other departments implementing surgeon-performed ultrasound across varying levels of operator experience [12,13,25,26]. Furthermore, the study is strengthened by using histopathology as the gold standard, supplemented by a six-month follow-up to minimize false-negative US-FNAB results.

This study has several limitations, including a relatively small sample size and its retrospective design. As the study reflects the standard of care in our clinic, no control group was included. Moreover, samples were collected by multiple surgeons with varying levels of experience, and cytology reports were generated by multiple pathologists, potentially introducing inter-practitioner bias in both the sampling method and analysis. Additionally, not all patients had final histopathological confirmation; however, follow-up indicated that patients with benign cytology did not develop malignancy within six months after biopsy.

The proportion of inadequate samples found in this study, 17/155 (11%), was comparable to a previously published review reporting inadequacy rates on 9.6% (95% CI 6.8-12.8) for radiologists and 11.0% (95% CI 8.4-14.0) for clinicians, respectively [14]. Other studies exploring surgeon-performed US-FNAB found inadequacy rates ranging from 8% to 34% [8,24]. These findings add to the growing evidence that surgeon-performed US-FNAB can achieve diagnostic performance comparable to that of radiologist-performed procedures. The rate of inadequate US-FNAB was similar between residents and consultants, in contrast to another study that found that experienced consultants had significantly better thyroid US-FNAB results than residents [27]. However, the slightly higher rate of inadequate procedures among consultants may reflect their greater exposure to diagnostic difficult cases such as venous malformations and other technically challenging lesions, as three of the inadequate cases are due to venous malformations.

The low ROM among samples categorized as suspected malignant was mainly attributable to salivary gland lesions. These lesions were frequently reported as Milan categories IVB (salivary gland neoplasm of uncertain malignant potential) and V (suspicious of malignancy) in the US-FNAB cytology results, but were subsequently proven benign on histopathology. In such cases, US-guided core needle biopsy (US-CNB) may provide greater diagnostic precision and help reduce false-positive rates in salivary gland tumors, with a reported specificity of 85-99% [28,29].

Although we found promising results with the implementation of surgeon-performed US-FNAB, larger studies with longer inclusion periods are necessary to increase statistical power and better capture learning curves among surgeons with varying levels of experience. In addition to diagnostics, US-guided interventions are increasingly used for minimally invasive treatments such as radiofrequency ablation (RFA) for thyroid nodules [30]. Further research should also focus on improving resident training in US-guided interventions and on developing validated tools to assess sampling competence [16,17,25].

## Conclusions

Our study demonstrates that surgeon-performed US-FNAB with same-day cytology effectively enables rapid diagnosis of head and neck lesions. US-FNABs conducted in the fast-track outpatient clinic achieved high adequacy rates and a short time to a diagnostic plan, with 97.4% patients receiving their cytology diagnosis at the initial visit. Compared with previous studies, our findings suggest that surgeon-performed US-FNAB and same-day cytology results may help reduce diagnostic delays in HNC. Additionally, ENT residents and consultants attained comparable adequate sampling rates, supporting the feasibility of incorporating US-FNAB into routine ENT practice across various experience levels.

## Appendices

**Table 4:** The definition and explanation behind the diagnostic categories: (1) Malignant, (2) Suspicious for malignancy, (3) Diagnostically undetermined and (4) Benign.

Diagnostic categories	Description
The malignant category	Includes squamous cell carcinoma, adenocarcinoma, carcinoma not otherwise specified, melanoma, lymphoma, and malignant tumor cells not otherwise specified.
The suspicious for malignancy category	Is a diagnostic category that is familiar to all cytologists. Samples are characterized by marked atypia, which are not sufficient for a definitive diagnosis of malignancy. It is used for cases with neoplastic features where a distinction cannot be made between a benign neoplasm and a malignant neoplasm and in some cases, reactive changes based on cytomorphologic findings.
The diagnostically undetermined category	Included lesions of undecided morphology, atypical cells and cystic lesions, that lack qualitative and/or quantitative cytomorphologic features required for a definitive diagnostic category, but where further investigation is recommended as the material raises suspicion of a specific diagnosis. This category includes follicular neoplasm in thyroid specimens.
The benign category	Encompasses a broad spectrum of changes, including both benign neoplasia and non-neoplastic changes. Benign neoplasia will include two commonly encountered benign salivary gland neoplasms: pleomorphic adenoma and Warthin tumor, but also, e.g., pilomatrixoma. The non-neoplastic subgroup encompasses a broad spectrum of lesions, including those related to inflammatory, hyperplastic, and reactive conditions. Importantly, these cases lack atypia or other features that might suggest a neoplastic process.
